# Pathogens in the bay: environmental *Staphylococcus saprophyticus* strains mirror clinical counterparts in virulence, biofilm formation, antimicrobial resistance, pathogenicity, and phage susceptibility

**DOI:** 10.1007/s10482-026-02367-x

**Published:** 2026-06-28

**Authors:** Isabella Monteiro Carvalho, Erica Cristina Soares e Silva, Geovana Lacerda Rodrigues, Natállia Duarte dos Santos, Raienne Vitória da Silva Soares, Monalessa Fábia Pereira, Ciro César Rossi, Marcia Giambiagi-deMarval

**Affiliations:** 1 Instituto de Microbiologia Paulo de Góes, Universidade Federal do Rio de Janeiro, Rio de Janeiro, RJ Brazil; 2 Departamento de Ciências Biológicas, Universidade do Estado de Minas Gerais, Carangola, MG Brazil; 3 Instituto de Biodiversidade e Sustentabilidade, Universidade Federal Do Rio de Janeiro, Macaé, RJ Brazil; 4 Departamento de Biologia Celular E Molecular, Universidade Federal Fluminense, Niterói, RJ Brazil

**Keywords:** *Staphylococcus saprophyticus*, Environmental reservoirs, Antimicrobial resistance, Biofilm, Phage therapy

## Abstract

*Staphylococcus saprophyticus* is a leading cause of urinary tract infections, yet little is known about its environmental reservoirs and traits outside clinical settings. This study aimed to compare clinical and environmental *S. saprophyticus* strains isolated from a polluted coastal ecosystem in Brazil, assessing their virulence-associated traits, antimicrobial resistance, biofilm formation, and susceptibility to bacteriophages. Forty strains (20 clinical, 20 environmental) were characterized using GTG_5_-PCR, virulence gene screening, antibiotic susceptibility testing, biofilm assays, exposure to sub-inhibitory concentrations of ciprofloxacin, *Tenebrio molitor* infection model, and phage activity tests. Genetic fingerprinting and virulence gene profiles revealed a high degree of similarity between environmental and clinical isolates, indicating the presence of shared virulence-associated determinants. Environmental strains exhibited resistance to multiple antibiotics and showed biofilm formation and larval survival patterns comparable to those observed for clinical isolates. Exposure to sub-inhibitory concentrations of ciprofloxacin increased biofilm formation in several strains. Bacteriophage CSF, originally isolated from swine farm effluent, displayed lytic and antibiofilm activity against a substantial proportion of the isolates. These findings highlight the presence of clinically relevant traits among environmental *S. saprophyticus* strains and underscore the importance of continued microbiological surveillance in anthropized aquatic environments.

## Introduction

*Staphylococcus saprophyticus* is a Gram-positive, catalase-positive, coagulase-negative bacterium commonly found in the skin, mucosal surfaces, and the human genitourinary tract (Paiva-Santos et al. 2018). Although traditionally regarded as a commensal microorganism, it has emerged as a significant opportunistic pathogen and is recognized as the second most frequent etiological agent of uncomplicated lower urinary tract infections (UTIs), particularly among young sexually active women (Djawadi et al. 2023). In addition to UTIs, this species has also been associated with other infections, including endocarditis, epididymitis, nephrolithiasis, prostatitis, and septicemia, particularly in immunocompromised individuals or patients with underlying conditions (Raz et al. 2005). Its ability to colonize and persist is associated with the expression of multiple virulence factors, including adhesins, enzymes (e.g. proteases and urease), and biofilm formation (Djawadi et al. 2023; Rafiee and Ghaemi 2023).

Despite the widespread environmental distribution of *S. saprophyticus*, most studies investigating its virulence mechanisms focus on clinical isolates (Lawal et al. 2021). Data regarding the pathogenic potential of aquatic strains remain scarce, particularly concerning the presence of virulence-associated genes, antimicrobial resistance profiles, and biofilm-forming capacity. This knowledge gap is especially concerning given the increasing evidence that aquatic environments impacted by human activities can serve as reservoirs and vectors of virulent and resistant microorganisms (Ahmad et al. 2025a), thereby contributing to the emergence of new public health threats.

Clinically, uncomplicated urinary tract infections caused by *S. saprophyticus* are commonly treated with first-line agents such as nitrofurantoin or trimethoprim–sulfamethoxazole, whereas fluoroquinolones, including ciprofloxacin, are typically reserved for more complicated infections or specific clinical scenarios (Hooton 2012; Mancuso et al. 2023). In both clinical and environmental settings, bacteria may frequently encounter sub-inhibitory concentrations of antibiotics due to incomplete treatments, pharmacokinetic fluctuations, or environmental contamination by antimicrobial residues. Such low antibiotic levels are known to trigger adaptive responses in bacteria, including modulation of biofilm formation and other persistence-related traits (Andersson and Hughes 2014).

Guanabara Bay, located in the metropolitan region of Rio de Janeiro, Brazil, is a heavily polluted coastal ecosystem, impacted by continuous discharges of domestic and industrial sewage, solid waste, and hospital effluents (Freire et al. 2023). The combination of intense urban and industrial activity with recreational and fishing use of these waters enhances human exposure to pathogenic microorganisms present in the environment (Canellas et al. 2025). In this context, the investigation of bacterial isolates from such waters represents a strategic approach for environmental monitoring and for assessing emerging threats.

Given this scenario, the present study aimed to compare the virulence profiles of aquatic and clinical *S. saprophyticus* strains through the analysis of virulence-associated genes, antimicrobial susceptibility, biofilm formation, and responses to subinhibitory concentrations of ciprofloxacin. In addition, the virulence of the strains was evaluated in an in vivo model using *Tenebrio molitor*. We also assessed the genetic diversity of the isolates and explored the potential of phage therapy as an alternative or complementary therapeutic strategy, considering the increasing antimicrobial resistance landscape and the limitations of conventional treatments.

## Materials and methods

### Microorganisms and culture conditions

This study included 40 *S. saprophyticus* strains (Table 1), comprising 20 isolates obtained from Guanabara Bay and 20 from patients treated in hospitals in the State of Rio de Janeiro. The aquatic isolates were previously collected between 2024 and 2025 at two distinct sites in Guanabara Bay, one near the shoreline (n = 16) and another approximately 10 km offshore (n = 4) (Vilar et al. 2025). Clinical isolates were previously obtained from patients presenting with urinary tract infections (n = 5), atopic dermatitis (n = 7), and skin colonization (n = 8), between 2018 and 2019 (Guimarães et al. 2024). Stocks were maintained at − 80 °C in Brain heart infusion (BHI) broth supplemented with 30% glycerol. Prior to the experiments, strains were reactivated on BHI agar plates at 37 °C for 24 h. Single colonies were then inoculated into BHI broth and incubated at 37 °C for 24 h under aerobic conditions to obtain fresh (overnight) cultures used in the subsequent assays.

**Table 1:** *Staphylococcus saprophyticus* strains used in this study

Strains	Isolation source	Reference
BG23, BG24, BG25, BG26, BG27, BG28, BG29, BG30, BG31, BG33, BG36, BG37, BG38, BG39, BG40, BG41	Guanabara bay, near the shoreline	(Vilar et al. 2025)
BG21, BG22, BG32, BG35	Guanabara bay, offshore	(Vilar et al. 2025)
60ad, 75c, 87c, 94c, 106c, 143c, 174c, 224c	Skin colonization	(Guimarães et al. 2024)
110ad, 228ad, 521ad, 601ad, 744ad, 759ad, 794ad	Atopic dermatitis	(Guimarães et al. 2024)
RC41, RC78a, RC141, RC181, RC299	Urinary tract infections	Unpublished^a^

^a^These strains were previously isolated from patients of the Antônio Pedro University Hospital of the Universidade Federal Fluminense (approved by the Research Ethics Committee of the School of Medicine at UFF, under nº 14,348,319.4.0000.5243)

## Assessing the genetic diversity of the strains

To evaluate the genetic diversity of the *Staphylococcus saprophyticus* strains used in this study, the GTG₅-PCR DNA fingerprinting technique (Švec et al. 2010) was employed. Briefly, genomic DNA was extracted from 1 mL of fresh bacterial culture using Chelex resin (Bio-Rad, USA), following the manufacturer's instructions. DNA integrity was werified by agarose gel electrophoresis and concentration determined by spectrophotometry. PCR reactions were performed with 100 ng of DNA, 1 µM GTG₅ primer (Table 2), and GoTaq™ Green Master Mix kit (Promega, USA). The thermal cycling consisted of denaturation at 95 °C for 35 s, annealing at 45 °C for 1 min, and extension at 72 °C for 5 min. A final extension step was performed at 72 °C for 5 min. PCR products were separated by electrophoresis on 1.2% agarose gels stained with ethidium bromide (0.5 µg/mL). The resulting banding patterns were analyzed using GelJ software (Heras et al. 2015), and a dendrogram was constructed based on the UPGMA (Unweighted Pair Group Method with Arithmetic Mean) clustering algorithm.

**Table 2:** Oligonucleotides used in this work

Target	Primer sequences (5’-3’)	Amplicon (bp)	Reference
GTG_5_	GTGGTGGTGGTGGTG	varied	De Vuyst et al. 2008
*aas*	GCCGACTACGCAGCAACTAAC CCATGAGGGTCAGAGTGGTCAG	136	Korte-Berwanger et al. 2013
*dsdA*	TTACTGAACCAACACATGCCCC ATTTGCCCGACTAAGCGAGATG	147	Korte-Berwanger et al. 2013
*sdrI*	CAACGTGCAACAACAGATGAC TATTTGATGGCGACGGAGTG	111	Paiva-Santos et al. 2018
*ssp*	TGGTGCTGCACATGCAGAAAG ACGGACAGTTTGTCCTCCCATAC	129	Korte-Berwanger et al. 2013
*sssF*	GGTACCAAAGTCTATTTGACG AGCTACCCGAGGAATCACC	118	Paiva-Santos et al. 2018
*uafA*	GTAGATGACTCCGTGGTTGAAG AGCGATTGTTCTCCCATTAGC	125	Paiva-Santos et al. 2018
*ureC*	CCATGTGTAATGGCTGGGTT GCAAGTTTGCTCACCGTATGA	122	Paiva-Santos et al. 2018

## Detection of virulence genes

Seven genes associated with *S. saprophyticus* virulence were investigated: *ssp* (surface-associated protein), *sdrI* (serine-aspartate repeat protein), *aas* (autolysin), *uafA* (uro-adherence factor A), *ureC* (urease), *dsdA* (D-serine deaminase), and *sssF* (surface protein). PCR reactions were performed using the GoTaq™ Green Master Mix kit (Promega, USA), according to the manufacturer’s recommendations. Each reaction contained 20 ng of genomic DNA and 0.2 µM of each primer (Table 2). The clinical isolate *S. saprophyticus* 7108 (Gatermann et al. 1992), which harbors all target genes, was used as the positive control. Thermal cycling conditions consisted of initial denaturation at 95 °C for 35 s, annealing at 50 °C for 30 s, and extension at 72 °C for 30 s, followed by a final extension at 72 °C for 5 min. PCR products were visualized by electrophoresis on 1.2% agarose gels stained with ethidium bromide (0.5 µg/mL).

## Antimicrobial susceptibility testing

Antimicrobial susceptibility was assessed using the disk diffusion method on Mueller–Hinton agar (Sigma-Aldrich, USA), following the guidelines of the Clinical and Laboratory Standards Institute (CLSI 2025). Bacterial suspensions were prepared from overnight cultures and adjusted to a 0.5 McFarland standard before being spread onto Mueller–Hinton agar plates. Antimicrobial disks (Oxoid, USA) included cefoxitin (30 μg), ciprofloxacin (5 μg), clindamycin (2 μg), erythromycin (15 μg), gentamicin (10 μg), linezolid (30 μg), mupirocin (200 μg), nitrofurantoin (300 μg), penicillin G (10 U), rifampicin (5 μg), sulfamethoxazole-trimethoprim (25 μg), and tetracycline (30 μg). All strains were specifically tested for methicillin resistance using the cefoxitin disk. Assays were performed in triplicate, and *S. aureus* ATCC 25923 was used as the quality control strain.

## Biofilm formation capacity of *S. saprophyticus* strains

Biofilm formation was evaluated using the crystal violet microtiter plate assay, as suggested by Barros et al. (2015). Aliquots of fresh overnight cultures were diluted 1:100 in BHI broth supplemented with 1% glucose, and 100 µL of the suspension were inoculated into polystyrene microtiter plate wells. Plates were incubated at 37 °C for 48 h to allow biofilm formation. After incubation, the supernatant was removed and the wells were gently washed with sterile saline solution to remove non-adherent cells. The plates were then dried and fixed at 60 °C for 30 min. Adherent cells were stained with 1% crystal violet, followed by washing and air drying. The remaining crystal violet was solubilized in 100 µL of ethanol, and absorbance was measured at 570 nm. Biofilm production was classified according to Stepanović et al. (2007) as non-producers (OD ≤ ODc), weak producers (ODc < OD ≤ 2 × ODc), moderate producers (2 × ODc < OD ≤ 4 × ODc), and strong producers (OD > 4 × ODc), where ODc corresponds to the mean optical density of the negative control plus three standard deviations.

*S. aureus* ATCC 35984, known for its strong biofilm-forming capacity under the tested conditions, was used as positive control, while wells containing sterile BHI medium only served as the negative control. All assays were performed in biological and technical triplicates.

## Effect of sub-inhibitory ciprofloxacin concentrations on biofilm formation

To evaluate the effect of sub-MIC ciprofloxacin on biofilm formation, six aquatic and six clinical *S. saprophyticus* strains were selected to represent the diversity of biofilm-forming phenotypes observed among the 40 strains. First, the minimum inhibitory concentration (MIC) of ciprofloxacin (Sigma-Aldrich, USA) were determined by broth microdilution following CLSI (2025) recommendations, using both Mueller–Hinton and BHI media for comparison. Bacterial suspensions were adjusted to a turbidity equivalent to 0.5 on the McFarland scale. *S. aureus* ATCC 29213 was used as the positive control, and wells containing only BHI broth served as the negative control. The selected strains were exposed to concentrations ranging from the MIC to MIC/16 in microtiter plates and incubated at 37 °C for 48 h. Biofilm quantification was performed by the crystal violet method, as described above.

## Survival assays in *Tenebrio molitor*

In vivo virulence of all strains was assessed in *Tenebrio molitor* larvae, as standardized by Andrade-Oliveira et al. (2023). *T. molitor* larvae with light and uniform coloration, weighing between 70 and 100 mg, were selected. *S. saprophyticus* strains were previously cultured in BHI broth for 24 h. Each larva was inoculated with 10 µL of a bacterial suspension containing approximately 10^6^ CFU using an insulin syringe into the last left proleg. Control groups received 10 µL of sterile saline solution (0.9% w/v NaCl). Following inoculation, larvae were placed in Petri dishes and incubated at 37 °C for 72 h. Mortality was monitored daily, and larvae were considered dead when no movement was observed in response to gentle stimulation or when signs of melanization were evident. Experiments were performed in groups of 15 larvae each, conducted in biological triplicates. The strains *Staphylococcus epidermidis* ATCC 35984, *Staphylococcus aureus* ATCC 29213, and the clinical isolate *S. saprophyticus* 7108 (Gatermann et al. 1992) were used as controls.

## Evaluation of *S. saprophyticus* susceptibility to the generalist phage CSF

The bacteriophage CSF, a broad-host-range phage capable of infecting multiple *Staphylococcaceae* species (Ahmad et al. 2025b), was evaluated for its lytic activity against the 40 *S. saprophyticus* strains. For propagation and maintenance, the phage was cultivated in its original host *Staphylococcus xylosus* PE2 (Ahmad et al. 2025a) and stored at 4 °C in SM buffer (0.1 M NaCl, 0.2 M MgSO_4_·7H_2_O, 1 M Tris–HCl, pH 7.5, and 0.1% gelatin). For lytic assays, 500 µL aliquots of exponentially growing bacterial cultures (~ 10^8^ CFU/mL) were mixed with 5 mL of BHI soft agar (0.7%) and overlaid onto BHI agar plates. After solidification, 3 µL drops of the CSF phage stock were spotted onto the plates, which were then incubated at 37 °C for 24 h. The formation of lysis zones was evaluated the following day and interpreted as indicative of phage-mediated cell lysis.

## Effects of phage CSF on biofilm formation

The antibiofilm activity of the bacteriophage CSF was evaluated against the 40 *S. saprophyticus* strains listed in Table 1, as suggested by Ahmad et al. (2026b). Exponentially growing bacterial cultures were co-incubated with different concentrations of the phage (10^8^–10^12^ PFU/mL) in BHI medium using 96-well polystyrene microtiter plates. After 48 h at 37 °C, biofilm biomass was quantified using the crystal violet assay described above. The strain PE2 (Ahmad et al. 2025a) without phage treatment was used as the positive control (100% biofilm formation), while wells containing only BHI served as the negative control. Biofilm reduction was expressed as a percentage. All experiments were performed in biological and technical triplicates.

## Statistical analysis

Statistical analyses were performed using GraphPad Prism 8 and SigmaPlot softwares. Survival data were analyzed using Kaplan–Meier survival curves and compared by the log-rank test (*p* < 0.05). Mean values of biofilm formation, with and without exposure to sub-MIC ciprofloxacin, were evaluated by ANOVA followed by Tukey’s multiple comparison test (*p* < 0.05). The effect of phage treatment on biofilm formation was assessed using Student’s *t*-test.

## Results

### Environmental and clinical *S. saprophyticus* strains are genetically closely related

**Fig. 1:**
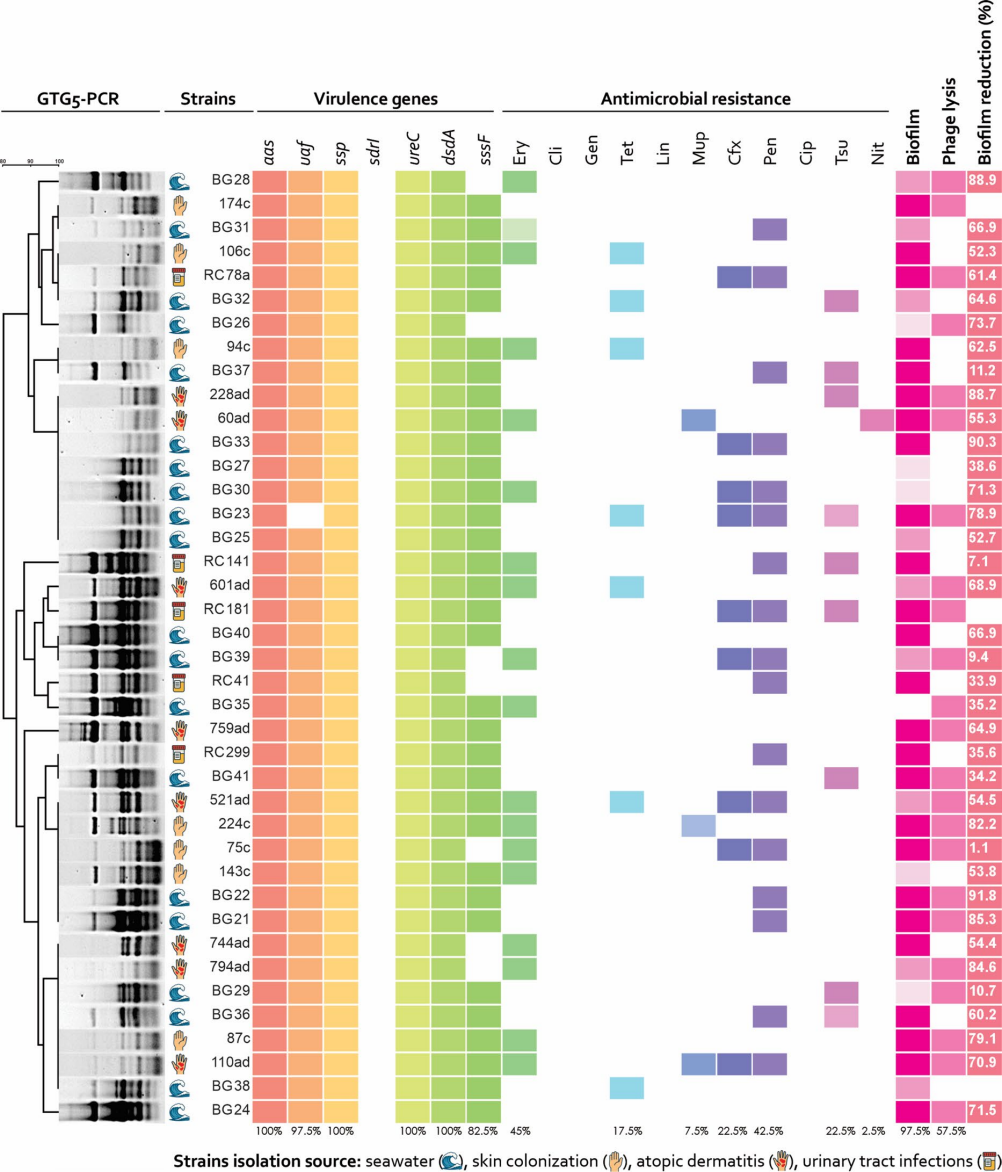
Overview of the main characteristics of *Staphylococcus saprophyticus* strains analyzed in this study, including genetic profiles assessed by GTG_5_-PCR, presence of virulence genes, antimicrobial resistance phenotypes, biofilm formation capacity, susceptibility to phage CSF, and biofilm reduction in the presence of the phage. Strain origins are indicated to the left of each name. Colored rectangles represent positive results for the corresponding feature. Lighter shades of the same color within a column indicate intermediate antibiotic susceptibility or graded levels of biofilm formation, classified as strong, moderate, or weak

The genetic profiles obtained through GTG_5_-PCR fingerprinting revealed a high degree of relatedness among the *S. saprophyticus* strains analyzed in this study, regardless of their origin—whether environmental (isolated from seawater) or clinical (urinary tract infections, atopic dermatitis, or skin colonization) (Fig. 1).

According to the clustering analysis, all strains shared at least 80% similarity, and several formed clusters with ≥ 90% similarity. Notably, multiple pairs of strains with higly similar fingerprinting profiles despite being isolated from distinct sources. Examples include BG40 and BG93; 87C and 110AD; BG33, 228AD, and 60AD; as well as BG23 and BG25, suggesting a close genetic relationship between environmental and clinical isolates.

## Virulence genes are widespread in *S. saprophyticus*, but antimicrobial resistance is more pronounced among clinical isolates

All *S. saprophyticus* strains analyzed carried the *aas* (autolysin/adhesin), *ssp* (surface-associated protein), *ureC* (urease), and *dsdA* (D-serine deaminase) genes, indicating a conserved core of virulence-associated factors (Fig. 1). The *uafA* gene, encoding a uro-adherence factor, and *sssF*, which encodes a surface-associated protein involved in host interaction, were also highly prevalent, detected in 97% and 82% of the isolates, respectively. One environmental strain lacked *uafA*, while *sssF* was absent in three aquatic and four clinical strains. The *sdrI* gene, which encodes a serine–aspartate repeat surface protein, was not detected in any of the samples.

In contrast to the broadly distributed virulence determinants, antimicrobial resistance was more prevalent among clinical isolates. Notably, resistance to erythromycin was observed in 65% of clinical strains compared to just 20% of environmental ones. Resistance rates for penicillin G were similarly high in both groups (40% in clinical and 45% in aquatic strains). Cefoxitin (25% vs. 20%), tetracycline (20% vs. 15%), and mupirocin (10% only among clinical isolates) also showed higher resistance levels in the clinical group. Interestingly, resistance to sulfamethoxazole-trimethoprim was slightly more frequent among environmental strains (20% vs. 15%). All strains were susceptible to clindamycin, gentamicin, linezolid, and ciprofloxacin.

## Biofilm formation is common among *S. saprophyticus* strains, with stronger adherence observed in clinical isolates

Biofilm production was observed in 97.5% of the *S. saprophyticus* strains (Fig. 1). Among clinical isolates, 75% were classified as strong biofilm producers, 20% as moderate, 5% as weak. In contrast, among environmental strains, 45% were strong producers, 25% moderate, 25% weak, and 5% non-producers.

Although strong biofilm formation was more frequently observed in clinical strains, no statistically significant difference was found in the mean optical density values between the two groups (*p* > 0.05).

## Sub-inhibitory concentrations of ciprofloxacin promote strain-dependent biofilm enhancement in *S. saprophyticus*

All *S. saprophyticus* strains analyzed were susceptible to ciprofloxacin, with MIC values ranging from 0.25 μg/mL to 0.5 μg/mL. To investigate the effects of sub-inhibitory concentrations (sub-MIC) on biofilm formation, selected strains—chosen to represent diverse adherence profiles and isolation sources—were exposed to ciprofloxacin at concentrations ranging from 1/2 to 1⁄16 of their respective MICs.

**Fig. 2:**
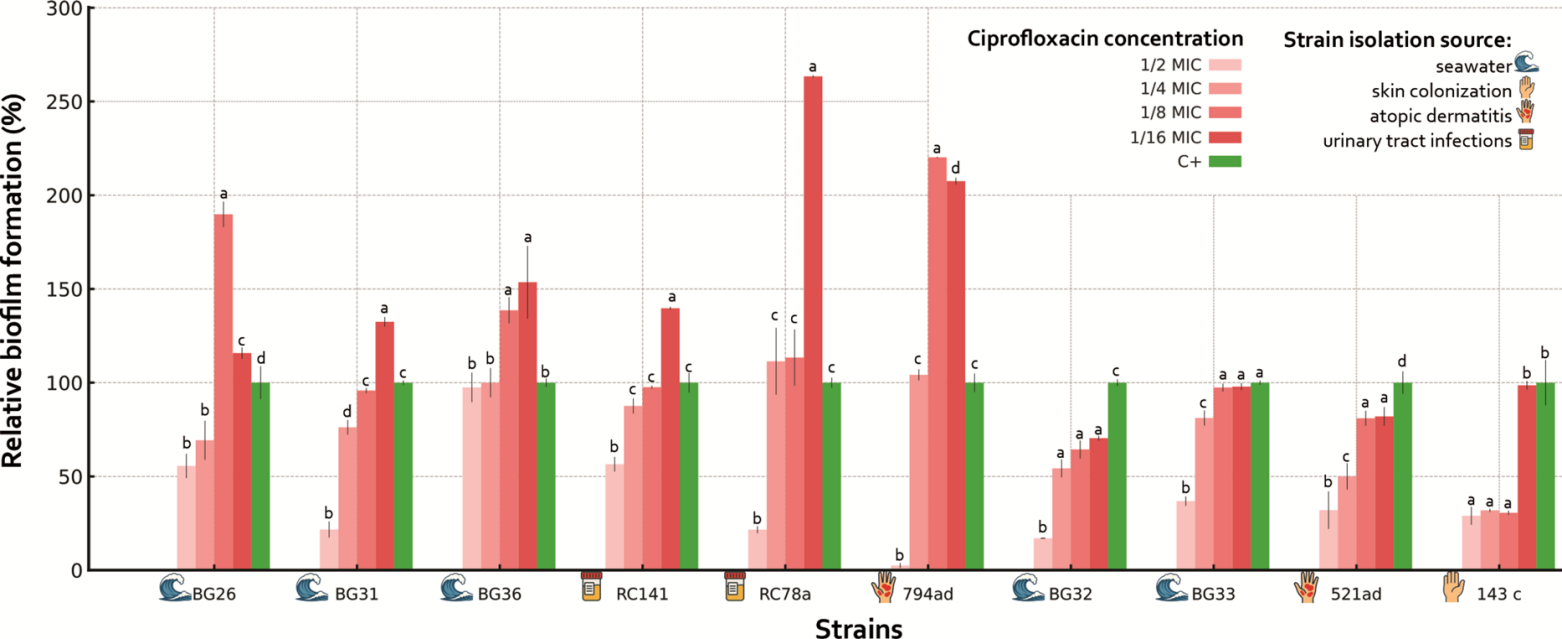
Biofilm formation by selected *Staphylococcus saprophyticus* strains in the presence of sub-inhibitory concentrations of ciprofloxacin. For comparison purposes, the biofilm production of each strain under control conditions (absence of antibiotic) was normalized to 100%. Bars represent mean ± SD. Different letters indicate statistically significant differences among treatments for the same strain according to ANOVA followed by Tukey’s multiple comparison test (*p* < 0.05)

The responses were heterogeneous across isolates. Although a general trend of increased biofilm formation with decreasing antibiotic concentration was observed, some strains showed particularly marked responses. In strains BG26, BG31, BG36, RC141, RC78a, and 794ad, exposure to concentrations between 1⁄8 and 1⁄16 MIC significantly enhanced biofilm production, often surpassing the levels observed in the untreated control for the same strain (Fig. 2).

Notably, strain BG26, originally classified as a weak biofilm producer, showed an 89% increase in biofilm formation at 1⁄8 MIC. In strains RC78a and 794ad, the increase exceeded 100%, underscoring the capacity of sub-inhibitory ciprofloxacin concentrations to enhance virulence-associated phenotypes in a strain-dependent manner.

## Environmental and clinical *S. saprophyticus* strains exhibit similar virulence in the *Tenebrio molitor* infection model

**Fig. 3:**
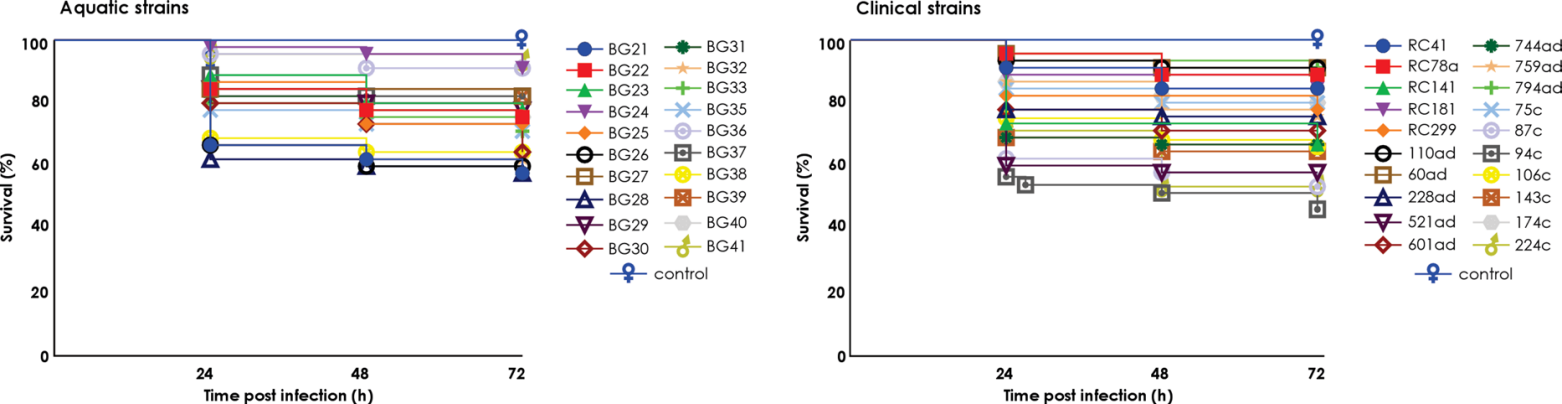
Survival curves of *Tenebrio molitor* larvae infected with environmental and clinical *Staphylococcus saprophyticus* strains over a 72-h post-infection period

Survival curves for all *S. saprophyticus* strains included in this study were evaluated using the alternative in vivo infection model *Tenebrio molitor*. Kaplan–Meier survival analysis revealed similar patterns between clinical and environmental groups, with all larvae inoculated at the same starting concentration of 10^6^ CFU/mL. In both groups, larval mortality occurred predominantly within the first 24 h post-infection. Notably, more than 50% of the larvae survived throughout the experimental period for all tested strains. No statistically significant differences were observed between the clinical and environmental isolates (log-rank test, *p* > 0.05) (Fig. 3).

## Phage CSF shows broad lytic activity and antibiofilm potential against *S. saprophyticus* strains

The lytic activity of bacteriophage CSF was assessed through host range assays, revealing variability in lysis efficiency across clinical and environmental *S. saprophyticus* strains (Fig. 1). Among environmental isolates, CSF lysed 50% of the strains, while in the clinical group, its activity was slightly higher, reaching 65%.

**Fig. 4:**
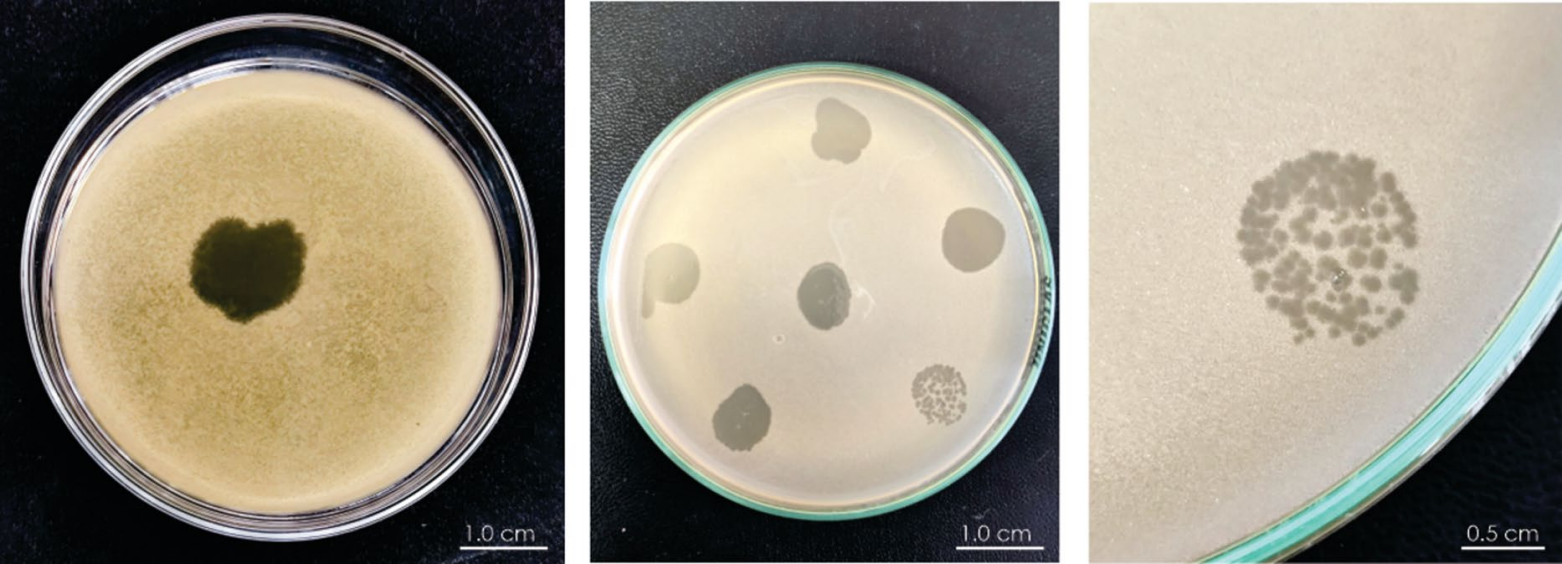
Representative image of bacteriophage CSF lytic activity against *Staphylococcus saprophyticus* BG21. Clear zones indicate areas of bacterial growth inhibition caused by viral lysis. The image on the right shows the formation of well-defined lysis plaques resulting from phage infection

Beyond its lytic action (Fig. 4), phage CSF also demonstrated significant antibiofilm effects, reducing biofilm formation in 95% (n = 19) of the environmental isolates and 90% (n = 18) of the clinical ones—even in strains where no lytic activity was detected. The maximum reduction observed was 91%, underscoring the phage’s potential to modulate biofilm formation and its promising application for controlling *S. saprophyticus* infections in both clinical and environmental contexts.

## Discussion

*Staphylococcus saprophyticus* is a well-recognized cause of uncomplicated urinary tract infections, particularly among young women (Djawadi et al. 2023) However, the ecology and circulation of this species outside clinical settings remain incompletely understood, especially in anthropogenically impacted aquatic environments. In this context, the high genetic similarity observed between environmental and clinical isolates in our study suggests that closely related *S. saprophyticus* lineages may circulate across environmental and human-associated niches. Although GTG_5_-PCR provides lower phylogenetic resolution than genome-based approaches such as cgMLST or SNP analyses, it remains a useful tool for rapidly assessing genetic relatedness and identifying major similarity patterns among isolates (Ahmad et al. 2025a). Such findings support the hypothesis that polluted aquatic environments may act as reservoirs for strains carrying traits relevant to human infection.

The detection of conserved virulence genes in nearly all strains—regardless of origin— indicates that environmental and clinical *S. saprophyticus* isolates share a common set of traits associated with host interaction. Although the virulence genes investigated here are chromosomally encoded, future studies exploring plasmid-associated virulence determinants could provide additional insights into the genetic basis of pathogenicity in this species. In particular, the presence of *dsdA* and *ureC*, genes previously linked to adaptation to the urinary tract, was observed in both groups of isolates (Gatermann et al. 1992; Rafiee and Ghaemi 2023). Although the presence of these genes alone does not demonstrate pathogenic potential, their conservation across environmental and clinical strains highlights the occurrence of virulence-associated determinants in isolates recovered from polluted aquatic environments.

While antimicrobial resistance was more pronounced in clinical isolates, environmental strains also exhibited resistance to multiple antibiotic classes, particularly β-lactams and sulfonamides. In contrast, resistance to mupirocin was not detected among environmental isolates, which may reflect the limited environmental exposure to this antibiotic, as it is primarily used as a topical agent in clinical settings (Dadashi et al. 2020). These results are consistent with previous reports from Guanabara Bay (Vilar et al. 2025) and highlight a recurring pattern: environmental strains of *Staphylococcus* not only survive but also adapt under selective pressure from antimicrobial residues present in coastal waters. This scenario underscores the role of urban water systems as both reservoirs and amplifiers of resistant bacteria.

Biofilm formation emerged as a nearly ubiquitous trait among isolates and may be a key factor facilitating the persistence of *S. saprophyticus* in both clinical and environmental settings. The frequent detection of strong biofilm phenotypes among clinical strains aligns with their role in recurrent UTIs and device-associated infections (Nguyen et al. 2020; Kranjec et al. 2021). This pattern may reflect selective pressures encountered during host colonization, where biofilm formation contributes to persistence, tolerance to antimicrobial exposure, and protection against host immune defenses. However, the presence of moderate to strong biofilm producers among aquatic isolates indicates that environmental strains are also capable of forming robust surface-attached communities—an important trait for survival in fluctuating, nutrient-limited, or toxic aquatic environments (Lawal et al. 2021; Rafiee and Ghaemi 2023).

The finding that ciprofloxacin, at sub-inhibitory concentrations, enhanced biofilm formation in several strains from both groups raises important clinical and ecological considerations. Sub-MIC exposure is likely in vivo during inappropriate dosing, poor pharmacokinetics, or in biofilm-associated infections (Elawady et al. 2024). In the environment, these concentrations may result from wastewater contamination (Sanchez-Cid et al. 2023; Wu et al. 2025). For instance, the predicted no-effect concentration (PNEC) proposed for ciprofloxacin in aquatic environments is approximately 0.064 µg/L, suggesting that even very low environmental concentrations may exert selective pressure on microbial populations (Bengtsson-Palme and Larsson 2016). The observed increase in biofilm formation suggests that ciprofloxacin exposure at sub-inhibitory levels may promote persistence rather than bacterial clearance. These data echo findings in methicillin-resistant *S. aureus* (MRSA) and uropathogenic *E. coli* (Rafaque et al. 2020; Park et al. 2024) and highlight the double-edged nature of fluoroquinolone use in infection management and its environmental footprint.

In vivo testing using *T. molitor* larvae confirmed that clinical and environmental isolates had similar virulence profiles under controlled infection conditions. These results suggest that environmental *S. saprophyticus* strains may retain pathogenic traits commonly associated with clinical isolates. The use of the *T. molitor* model provided a practical approach for comparing virulence across strains and contributes to the growing use of invertebrate systems for studying emerging environmental pathogens (Ngoc Chau and Tuan Anh 2022; Andrade-Oliveira et al. 2023).

Phage therapy has re-emerged as a promising alternative or adjunct to antibiotics, particularly in the fight against multidrug-resistant biofilm-forming pathogens (Kortright et al. 2019; Górski et al. 2020). In this study, phage CSF showed lytic activity against a substantial proportion of *S. saprophyticus* isolates, demonstrating a broad host range, suitable for potential combination in phage-based therapeutic approaches. Previous analyses demonstrated that CSF is a strictly lytic phage lacking known virulence or antibiotic resistance genes, supporting its suitability for further exploration as a biocontrol agent against staphylococcal strains (Ahmad et al. 2025b).

Beyond lytic activity, phage CSF also exhibited pronounced antibiofilm effects, including against strains that were not susceptible to direct lysis. These effects are consistent with previous reports (Viana et al. 2025) demonstrating that phages can interfere with biofilms through enzymatic degradation of matrix components such as exopolysaccharides, proteins, and nucleic acids (Pires et al. 2017; Ahmad et al. 2026a). The dual lytic and antibiofilm potential of phage CSF reinforces the promise of phage therapy as a complementary strategy for controlling *S. saprophyticus* infections in both clinical and environmental contexts. The slightly higher susceptibility observed among clinical isolates may reflect differences in surface receptors required for phage adsorption or the presence of phage-defense systems in environmental strains, which are frequently exposed to diverse viral populations (Breitbart 2012).

In conclusion, this study provides compelling evidence that *S. saprophyticus* strains isolated from a recreational aquatic environment can harbor virulence and resistance features comparable to those of clinical strains. The genetic similarity, conserved virulence gene profiles, biofilm-forming ability, and equivalent virulence in the *T. molitor* model strongly support the notion that environmental isolates should not be underestimated as potential human pathogens. These findings underscore the critical importance of microbiological surveillance in anthropized ecosystems. Moreover, research into alternative control strategies, such as phage therapy, is essential to reduce the public health risks posed by these environmental reservoirs.

## Data Availability

All data are included in the manuscript.

## References

[CR1] Ahmad F, Martuchelle SS, Andrade-Oliveira AL et al (2025a) From farm to community: dispersal of potentially pathogenic *Staphylococcus* and *Mammaliicoccus* species and antimicrobial resistance across shared environments. Curr Microbiol 82:104. 10.1007/s00284-025-04079-3 39875692 10.1007/s00284-025-04079-3

[CR2] Ahmad F, Garcia PR, Viana VEL et al (2026b) pTJK, a rare *Mammaliicoccus lentus* phage with broad-host-range, antibiofilm, and synergistic interactions with antimicrobials against resistant Staphylococcaceae. Arch Microbiol 208:243. 10.1007/s00203-026-04786-w 41805917 10.1007/s00203-026-04786-w PMC12975815

[CR3] Ahmad F, Viana VEL, de Rezende RR, et al (2025b) Discovery of phage CSF, a novel generalist bacteriophage targeting multidrug-resistant and potentially pathogenic *Staphylococcus* spp. and *Mammaliicoccus* spp. Arch Virol. 10.1007/s00705-025-06370-x 10.1007/s00705-025-06370-x 40731165

[CR4] Ahmad F, Garcia P, Viana V, et al (2026a) pTJK, a rare *Mammaliicoccus lentus* phage with broad-host-range, antibiofilm, and synergistic interactions with antimicrobials against resistant *Staphylococcaceae* . Arch Microbiol 2026:in press 10.1007/s00203-026-04786-w PMC12975815 41805917

[CR5] Andersson DI, Hughes D (2014) Microbiological effects of sublethal levels of antibiotics. Nat Rev Microbiol 12:465–478. 10.1038/nrmicro3270 24861036 10.1038/nrmicro3270

[CR6] Andrade-Oliveira AL, Lacerda-Rodrigues G, Pereira MF et al (2023) *Tenebrio molitor* as a model system to study *Staphylococcus* spp virulence and horizontal gene transfer. Microb Pathog 183:106304. 10.1016/j.micpath.2023.106304 37567328 10.1016/j.micpath.2023.106304

[CR7] Barros EM, Lemos M, Souto-Padrón T, Giambiagi-deMarval M (2015) Phenotypic and genotypic characterization of biofilm formation in *Staphylococcus haemolyticus*. Curr Microbiol 70:829–834. 10.1007/s00284-015-0794-x 25724344 10.1007/s00284-015-0794-x

[CR8] Bengtsson-Palme J, Larsson DGJ (2016) Concentrations of antibiotics predicted to select for resistant bacteria: proposed limits for environmental regulation. Environ Int 86:140–149. 10.1016/j.envint.2015.10.015 26590482 10.1016/j.envint.2015.10.015

[CR9] Breitbart M (2012) Marine viruses: truth or dare. Ann Rev Mar Sci. 10.1146/annurev-marine-120709-142805 22457982 10.1146/annurev-marine-120709-142805

[CR10] Canellas ALB, de Oliveira BFR, Laport MS (2025) Unveiling pathogenicity and resistance profiles of non-cholera *Vibrio* from an urban estuary. Braz J Microbiol 56:1631–1640. 10.1007/s42770-025-01700-2 40399596 10.1007/s42770-025-01700-2 PMC12350869

[CR11] CLSI (2025) Performance Standards for Antimicrobial Susceptibility Testing. Document M100 35th edition:ED35:2025

[CR12] Dadashi M, Hajikhani B, Darban-Sarokhalil D et al (2020) Mupirocin resistance in *Staphylococcus aureus*: A systematic review and meta-analysis. J Glob Antimicrob Resist. 10.1016/j.jgar.2019.07.032 31442624 10.1016/j.jgar.2019.07.032

[CR13] De Vuyst L, Camu N, De Winter T et al (2008) Validation of the (GTG)5-rep-PCR fingerprinting technique for rapid classification and identification of acetic acid bacteria, with a focus on isolates from Ghanaian fermented cocoa beans. Int J Food Microbiol 125:79–90. 10.1016/j.ijfoodmicro.2007.02.030 17920717 10.1016/j.ijfoodmicro.2007.02.030

[CR14] Djawadi B, Heidari N, Mohseni M (2023) UTI caused by *Staphylococcus saprophyticus*. In: Hegazy W Urinary Tract Infections IntechOpen Ch.2–142p. 10.5772/intechopen.110275

[CR15] Elawady R, Aboulela AG, Gaballah A et al (2024) Antimicrobial Sub-MIC induces *Staphylococcus aureus* biofilm formation without affecting the bacterial count. BMC Infect Dis 24:1065. 10.1186/s12879-024-09790-3 39342123 10.1186/s12879-024-09790-3 PMC11438285

[CR16] Freire MM, Gomez C, Moreira JC, Linde Arias AR (2023) Multibiomarker approach in fish to assess a heavily polluted Brazilian estuary, Guanabara Bay. Environ Monit Assess 195:187. 10.1007/s10661-022-10752-y 10.1007/s10661-022-10752-y 36504393

[CR17] Gatermann S, Kreft B, Marre R, Wanner G (1992) Identification and characterization of a surface-associated protein (Ssp) of *Staphylococcus saprophyticus*. Infect Immun 60:1055–1060. 10.1128/iai.60.3.1055-1060.1992 1541520 10.1128/iai.60.3.1055-1060.1992 PMC257593

[CR18] Górski A, Międzybrodzki R, Węgrzyn G et al (2020) Phage therapy: current status and perspectives. Med Res Rev 40:459–463. 10.1002/med.21593 31062882 10.1002/med.21593

[CR19] Guimarães LC, Garcia GD, Cavalcante FS, et al (2024) Methicillin-resistant *Staphylococcus aureus* and coagulase-negative *Staphylococcus* produce antimicrobial substances against members of the skin microbiota in children with atopic dermatitis. FEMS Microbiol Ecol 100:fiae070. 10.1093/femsec/fiae070 10.1093/femsec/fiae070 PMC11141783 38806244

[CR20] Heras J, Domínguez C, Mata E et al (2015) GelJ-a tool for analyzing DNA fingerprint gel images. BMC Bioinformatics 16:270. 10.1186/s12859-015-0703-0 26307353 10.1186/s12859-015-0703-0 PMC4549892

[CR21] Hooton TM (2012) Uncomplicated urinary tract infection. N Engl J Med 366:1028–1037. 10.1056/NEJMcp1104429 22417256 10.1056/NEJMcp1104429

[CR22] Korte-Berwanger M, Sakinc T, Kline K et al (2013) Significance of the *d* -Serine-Deaminase and *d* -Serine Metabolism of *Staphylococcus saprophyticus* for Virulence. Infect Immun 81:4525–4533. 10.1128/IAI.00599-13 24082071 10.1128/IAI.00599-13 PMC3837983

[CR23] Kortright KE, Chan BK, Koff JL, Turner PE (2019) Phage therapy: a renewed approach to combat antibiotic-resistant bacteria. Cell Host Microbe 25:219–232. 10.1016/j.chom.2019.01.014 30763536 10.1016/j.chom.2019.01.014

[CR24] Kranjec C, Morales Angeles D, Torrissen Mårli M et al (2021) Staphylococcal biofilms: challenges and novel therapeutic perspectives. Antibiotics 10:131. 10.3390/antibiotics10020131 33573022 10.3390/antibiotics10020131 PMC7911828

[CR25] Lawal OU, Fraqueza MJ, Bouchami O et al (2021) Foodborne origin and local and global spread of *Staphylococcus saprophyticus* causing human urinary tract infections. Emerg Infect Dis 27:880–893. 10.3201/eid2703.200852 33622483 10.3201/eid2703.200852 PMC7920669

[CR26] Mancuso G, Midiri A, Gerace E et al (2023) Urinary tract infections: the current scenario and future prospects. Pathogens 12:623. 10.3390/pathogens12040623 37111509 10.3390/pathogens12040623 PMC10145414

[CR27] Ngoc Chau N, Tuan Anh D (2022) The virulence and reproduction of six entomopathogenic nematode strains of species *Steinernema longicaudum* to mealworm *Tenebrio molitor* in the laboratory conditions. Vietnam J Biotech. 10.15625/1811-4989/15675

[CR28] Nguyen HTT, Nguyen TH, Otto M (2020) The staphylococcal exopolysaccharide PIA–Biosynthesis and role in biofilm formation, colonization, and infection. Comput Struct Biotechnol J 18:3324–3334. 10.1016/j.csbj.2020.10.027 33240473 10.1016/j.csbj.2020.10.027 PMC7674160

[CR29] Paiva-Santos W, Sousa VS, Giambiagi-deMarval M (2018) Occurrence of virulence-associated genes among *Staphylococcus saprophyticus* isolated from different sources. Microb Pathog 119:9–11. 10.1016/j.micpath.2018.03.054 29604423 10.1016/j.micpath.2018.03.054

[CR30] Park K-H, Kim D, Jung M et al (2024) Effects of sub-inhibitory concentrations of nafcillin, vancomycin, ciprofloxacin, and rifampin on biofilm formation of clinical methicillin-resistant *Staphylococcus aureus*. Microbiol Spectr 12:e0341223. 10.1128/spectrum.03412-23 38651875 10.1128/spectrum.03412-23 PMC11237638

[CR31] Pires D, Melo L, Vilas Boas D et al (2017) Phage therapy as an alternative or complementary strategy to prevent and control biofilm-related infections. Curr Opin Microbiol 39:48–56. 10.1016/j.mib.2017.09.004 28964986 10.1016/j.mib.2017.09.004

[CR32] Rafaque Z, Abid N, Liaqat N et al (2020) In-vitro investigation of antibiotics efficacy against uropathogenic *Escherichia coli* biofilms and antibiotic induced biofilm formation at sub-minimum inhibitory concentration of ciprofloxacin. Infect Drug Resist 13:2801–2810. 10.2147/IDR.S258355 32848429 10.2147/IDR.S258355 PMC7429215

[CR33] Rafiee M, Ghaemi EA (2023) Detection of virulence genes among *Staphylococcus saprophyticus* isolated from women with urinary tract infections: first report from Iran. BMC Res Notes 16:206. 10.1186/s13104-023-06481-1 37697340 10.1186/s13104-023-06481-1 PMC10496302

[CR34] Raz R, Colodner R, Kunin CM (2005) Who are you - *Staphylococcus saprophyticus*? Clin Infect Dis 40:896–898. 10.1086/428353 15736028 10.1086/428353

[CR35] Sanchez-Cid C, Ghaly TM, Gillings MR, Vogel TM (2023) Sub-inhibitory gentamicin pollution induces gentamicin resistance gene integration in class 1 integrons in the environment. Sci Rep 13:8612. 10.1038/s41598-023-35074-y 37244902 10.1038/s41598-023-35074-y PMC10224954

[CR36] Stepanović S, Vuković D, Hola V et al (2007) Quantification of biofilm in microtiter plates: overview of testing conditions and practical recommendations for assessment of biofilm production by staphylococci. APMIS 115:891–899. 10.1111/j.1600-0463.2007.apm_630.x 17696944 10.1111/j.1600-0463.2007.apm_630.x

[CR37] Švec P, Pantůček R, Petráš P, et al (2010) Identification of *Staphylococcus* spp. using (GTG)5-PCR fingerprinting. Syst Appl Microbiol. 10.1016/j.syapm.2010.09.004 10.1016/j.syapm.2010.09.004 21095086

[CR38] Viana VEL, Ahmand F, Martuchelle SS, et al (2025) From farm effluent to biotechnological potential: pGLS, a novel and resilient temperate bacteriophage with synergistic activity and broad antibiofilm properties against *Staphylococcus* and *Mammaliicoccus*. J Appl Microbiol lxaf118 10.1093/jambio/lxaf118 40359157

[CR39] Vilar LC, Rego ACS, Miguel MAL et al (2025) *Staphylococcus* spp. and methicillin-resistance gene *mecA* dispersion in seawater: a case study of Guanabara Bay’s recreational and touristic waters. Comp Immunol Microbiol Infect Dis 118:102326. 10.1016/j.cimid.2025.102326 39954386 10.1016/j.cimid.2025.102326

[CR40] Wu X, Sun H, Wang G et al (2025) Sub-inhibitory antibiotics reshape resistance dynamics in cyanobacteria-dominated waters. J Environ Sci. 10.1016/j.jes.2025.08.010 10.1016/j.jes.2025.08.010 42217863

